# Autophagy and Inflammatory Response in the Tumor Microenvironment

**DOI:** 10.3390/ijms18092016

**Published:** 2017-09-20

**Authors:** Daniel Ngabire, Gun-Do Kim

**Affiliations:** Department of Microbiology, College of Natural Sciences, Pukyong National University, 45, Yongso-ro, Nam-Gu, Busan 608-737, Korea; danyngabire@gmail.com

**Keywords:** autophagy, cancer, tumor microenvironment, tumor-associated macrophages, cancer-associated fibroblasts

## Abstract

Cell death is the last fate of the life cycle of cells. Different pathways involved in cell death are known to date, and are mostly represented by apoptosis, necrosis, and autophagy. Autophagy is one of the most preserved cell death pathways, characterized by the elimination of large parts of cytoplasmic components after being consumed by a double-membraned vesicle called an autophagosome. The formed autophagosome then fuses with a lysosome containing degrading enzymes and leads to the digestion of the autophagosome content. Autophagy is triggered by stress-related inducers, and is partially dependent on apoptotic proteins. It plays a major role in cancer, particularly in the tumor microenvironment where it has a paradoxical function in acting as a tumor suppressor and also as a tumor promoter. In the tumor microenvironment, autophagy regulates the differentiation of macrophages into tumor-associated macrophages (TAMs) and fibroblasts into cancer-associated fibroblasts (CAFs). TAMs and CAFs are abundantly present in the tumor microenvironment, and participate actively in tumor growth, tumor invasiveness, and tumor resistance to chemotherapy.

## 1. Introduction

**Figure 1:**
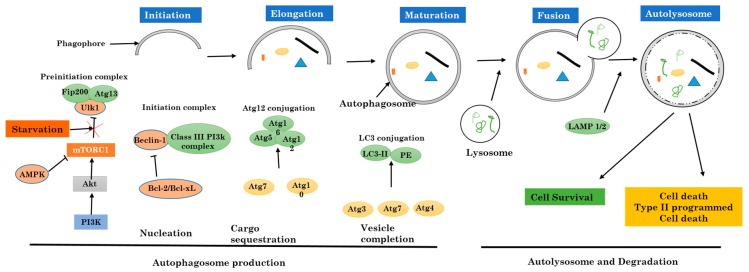
Activation and regulation of autophagy.

Autophagy, literally defined as self-eating, is a destructive cellular pathway that implicates the removal of encircled cytoplasmic cell components by lysosomes which contains degradant enzymes. To date, three forms of autophagy are known—chaperone-mediated autophagy, microautophagy, and macroautophagy. Both differ from each other due to their initiation, the mechanisms involved, and the mode of destruction during delivery to the lysosome [1,2] (Figure 1). Macroautophagy is the most referred to when autophagy is mentioned. Autophagy is commonly a survival process displayed when cells face stressful and abnormal conditions like elevated temperature, lack of sufficient nutrients, or low level of oxygen, therefore autophagy plays a crucial role in the maintenance of the cellular homeostasis [3,4]. More than being a guardian to cells, autophagy is also implicated in a significant number of human disorders like cancer, or neurodegenerative diseases like Parkinson’s and Alzheimer’s [5]. Autophagy is also implicated in immune system defense, both innate and adaptative. It is present during all different steps of inflammation, from the cellular level like macrophage differentiation and polarization, to molecular levels like the production of cytokines and various other inflammatory mediators. Autophagy plays an important role in the elimination of invaders like parasites, viruses, and bacteria, and additionally participates in the presentation of antigens by major histocompatibility complex (MHC) Class II molecules [6].

Cancer refers to cells that have lost control of the regulation of their cell cycle and had gained the ability to proliferate indefinitely. Cancer cells in tissue, after dividing, will develop into a mass of cells called a tumor [7]. Tumors produce many different molecules to increase their growth, to suppress the immune system defense, or to invade other organs.

Inside a tumor, a wide range of cells are present and work together with cancer cells to sustain and maintain a suitable environment for tumor progression. This environment is often referred to as the tumor microenvironment [8]. Immune cells, particularly macrophages are brought to this environment following the production of chemokines that allow their extravasation from blood vessels through the dilation of blood vessels layer.

In this review, we focus on the function of autophagy in cancer immunity, its interplay in the tumor microenvironment, and how it is a major process displayed during all events of tumor progression.

## 2. Autophagy

The process of autophagy is very important during cell death. It can be related to cell death but in most cases assists cell for survival by suppressing the initiation of apoptosis in cells. Increased autophagy in the absence of nutrients or cell growth factors triggers cell death escape [9,10] by the suppression of apoptosis. Cells can also be protected by autophagy via various other apoptotic inducers [11].

Autophagy is known to be a biological process or mechanism in which the intracellular (cytoplasmic) cell contents (organelles) are eliminated. It is the primary destructive mechanism for long-lived proteins, and therefore sustains a good quality control for proteins and cell organelles (Figure 1).

Initial steps in autophagy include the nucleation, elongation, and maturation of an isolated membrane usually called a phagophore. The ends of the formed phagophore then unite to form the autophagosome, which is a vesicle with double membranes that locks in specific targeted cytoplasmic components. After this step, the fusion of the autophagosome to a lysosome follows to form an autolysosome where the captured materials, both together with the inside membrane, are destroyed. 

### 2.1. Mechanism and Regulation of Autophagy

Inadequate autophagy can be deleterious [12,13], as can be its exorbitant activation; therefore autophagy in a cell must be strictly controlled. The induction together with the regulation of autophagy has been well investigated, mostly in yeast, in mammalian cells, and in Drosophila.

The formation and maturation of the autophagosome in the cytoplasm are controlled and regulated by a set of multiple proteins both related to autophagy (ATG proteins). The preinitiation complex is formed first, and is made of Unc-51-like kinase 1 (ULK1), FIP200 protein, and ATG13. Two major proteins regulate this complex: the mammalian target of rapamycin complex 1 (mTORC1) from the PI3k-Akt pathway which inhibits autophagy, and adenosine monophosphate (AMP)-activated protein kinase (AMPK) that inhibits mammalian target of rapamycin complex 1 (mTORC1) [14]. This step marks the autophagy initiation phase. In normal cells, the preinitiation complex is inhibited by mTORC1 through the phosphorylation of ULK1 and ATG13 proteins. With malfunction due to metabolic stress like shortages in amino acid, inhibition of ULK1 and ATG13 proteins by mTORC1 is suppressed [15,16]. In contrast, AMPK activates the preinitiation complex [17]. When ATP molecules are being consumed and not successfully replaced, AMP alongside with adenosine diphosphate (ADP) accumulate and activate AMPK. The activated AMPK initiates indirectly autophagy by suppressing the activity of mTORC1, which afterward allows the phosphorylation and activation of ULK1. Thus, the preinitiation complex plays a major role in the inhibition of mTORC1 and/or activation of AMPK (Figure 1).

The preinitiation complex attracts and then activates another initiative complex with beclin 1, a protein in the class III PI3K (Vps34), and a kinase protein Vps15. This complex formation results in the production of phosphatidylinositol 3-phosphate (PI3P) [18]. The activity of the initiation complex is negatively regulated by many different signaling pathways. Beclin-1 is instantly phosphorylated by Akt and later binds to 14-3-3 [19]. The obtained complex Beclin-1–14-3-3 is sealed with vimentin protein, which is a key component of the cell cytoskeleton intermediate filaments. Therefore, the sequestration of this complex mediated by the Akt pathway suppresses the kinase activity of the initiation complex. All this taken together prove that growth factors alongside with the PI3K-AKT pathway are able to prevent autophagy both directly and indirectly through Beclin-1 and mTOR pathway respectively. The Beclin-1 is a puzzling protein that needs many other joining units, not only Vps34 and Vps15, to regulate the preinitiation complex activities [20]. For instance, Beclin-1 links and interacts with autophagy/Beclin-1 regulator 1 (AMBRA1). AMBRA1 is a protein that attaches to the beclin-1–Vps34 complex in cytoskeleton microtubules via the dynein cytoskeletal proteins complex [21,22]. For autophagy induction, ULK1 phosphorylates AMBRA1 and then dissociates from the cell cytoskeleton, which permits the formation of the autophagosome.

In addition, the Bcl2 family proteins are capable of regulating the initiation complex via its specific BH3 domain. Beclin-1 is confined by antiapoptotic proteins like Bcl2 and Bcl-xL [23,24,25], and therefore inhibits the activity of the initiation complex. Under highly stressful conditions such as nutrient deprivation and low levels of oxygen, BH3-only protein holders like Bad and Bnip3 will free Beclin-1 and allow it to participate in the cell autophagy process [26,27]. In the other hand, the phosphorylation of the Beclin–1 BH3 domain of the death-associated protein kinase (DAPK) can lead to the separation of Beclin-1 from Bcl2 and Bcl-xL [28,29]. For the final step, protein kinases which belong to the c-Jun N-terminal kinases (JNK) family disrupt the complex through direct phosphorylation of Bcl2, therefore, diminishing its affinity for Beclin-1 [30,31]. All these previous steps are crucial for the control and supervision of autophagy during physical exercises and glucose metabolism in muscles.

The elongation phase and the late phase in the formation of autophagosomes are controlled by two separate but complementary ubiquitin like pathways: the ATG5-12 proteins and LC3-PE proteins. All these pathways start with a unique activating enzyme E1-ligase-like, ATG7, enrolled to the phagophore through PI3P from the initiation complex. At the beginning, ATG7, via its active cysteine residue, is strongly attached to a small protein ATG12 by a thioester bond. ATG12 is further transferred to another conjugating enzyme E2-like, ATG10, which later sends away ATG12 to ATG5. At the end, the ATG5-ATG12 complex interacts with ATG16L to create a larger multimeric complex needed to stabilize the forming phagophore [32]. 

The LC3-PE complex connection pathway starts after cleavage of LC3 by ATG4, also known to be a cysteine protease. The cleaved LC3 then interacts with ATG7 and is instantly shifted away to the E2-like enzyme ATG3. The previously formed complex ATG5-ATG12-ATG16L works as an E3 ligase, associating LC3 (Light chain 3) to phosphatidylethanolamine (PE) to form a LC3-PE complex (also called LC3-II) (Figure 1).

This newly formed complex, LC3-II, is very important for the fusion of autophagosomes with lysosomes. As soon as this step is terminated, the autophagosome fuses with lysosomes, and from the fusion, will result in one double membrane vesicle called autolysosome. The fusion procedure is terminated after membranes from both units are gathered together with the help of autophagosomal proteins syntaxin 17 (Stx17), SNAP29, and VAMP8. More interestingly, two known proteins that belong to lysosomes, LAMP1 and LAMP2, are needed for the fusion process, and when they are not available the autophagosome fails to fuse to lysosomes [33,34,35]. Inside the autolysosome, the trapped and engulfed cell components will be destroyed [36].

### 2.2. Autophagy Versus Apoptosis and Necrosis

Apoptosis or apoptotic cell death is the major part initiated by either the death receptors or by the mitochondrial pathway. Death receptors (DRs), for example, the Fas receptor which is also known as CD95, and the Trail receptor (TRAIL-R), or tumor necrosis factor receptor 1 (TNFR1) all induce apoptosis by directly recruiting and activating caspases which react with their respective ligands. The mitochondrial apoptotic pathway commences with signals from the damaged outside membrane of the mitochondrion after the release of apoptosis activating factors, particularly cytochrome c, into the cell cytosol. The mitochondrial apoptotic pathway is ruled by the Bcl2 protein family. When it enters the cytosol, cytochrome c induces the formation of a caspase activating complex named apoptosome. All of the above-cited pathways lead to the activation of caspase-specific proteases which then follows intracellular proteins cleavage, which in the end will lead to the destruction of the cell [26]. 

**Figure 2:**
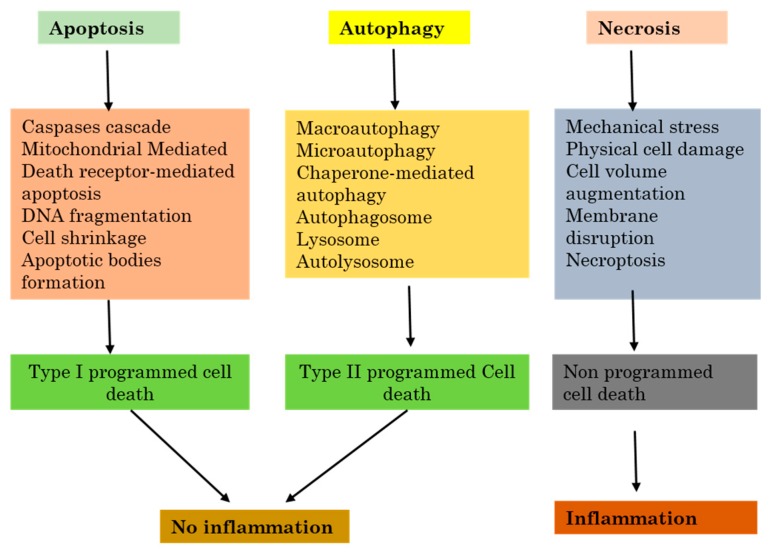
The relation between inflammation and the three major cell death pathways.

Necrosis also called necrotic cell death regroups a significant large variety of cell death paths that share in common their loss of the physiological structure of the cell cytoplasmic membrane to which follows the leakage cytoplasmic elements [37]. Necrosis can occur following many events which affect the cell homeostasis or repeated extensive damage, and both will result in cell death with loss of cell integrity. Some of the examples are elevated temperature, repeated freeze-thaw series, or during other stressful situations. In these cases, necrosis is passive because it does not need the activation of any specific protein pathways. The breakage of the cell cytoplasmic membrane can also be noticed in the late final steps of apoptotic or autophagic cell death processes, especially when phagocytosis does not successfully remove dead cells from circulation. As no signaling pathway is involved in this path, it is mainly called secondary necrosis, to differentiate it from other programmed death like apoptosis, necroptosis, or autophagy. Necrosis is not fully a result of pure hazard or passive processes, because it can regroup well-organized successive events (Figure 2).

One of the best most described forms of necrotic death is dependent on a protein kinase RIP, and is called “necroptosis” [38]. This process demands the kinase activity of RIP3, and can lead to a quick cell death with characteristics specific to necrosis [39,40,41]. Necroptosis can be engaged downstream from tumor necrosis factor receptor 1 (TNFR1) [42,43].

Furthermore, the catalytic activity of the caspase-8–FLIP heterodimer significantly inhibits the induction of RIP-dependent necrosis through a ripoptosome activated through TLR3 and TLR4 [44].

### 2.3. Autophagy in Cancer

**Figure 3:**
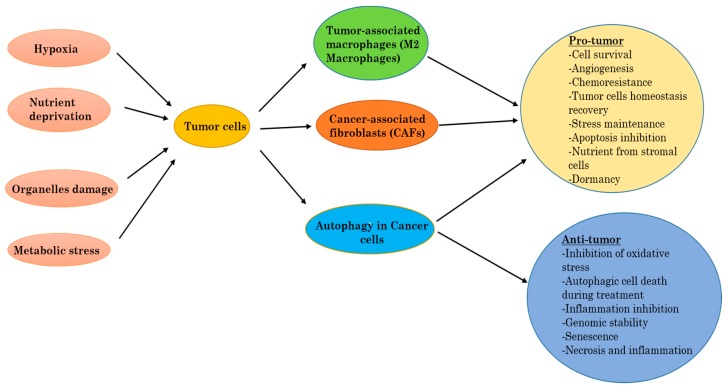
Autophagy, tumor-associated macrophages (TAMs), and cancer-associated fibroblasts (CAFs) into the tumor.

Autophagy can work to promote tumor cell survival [45], but can also participate in cell death [46]. The regulation of autophagy can either be elevated or suppressed by anticancer agents, and an increase of autophagy during cancer therapy can be in favor of survival (pro-survival), or induce cell death (pro-death) in tumor cells [47,48]. The precise and exact role that autophagy has in cancer is therefore dependent on the context, and we discuss in the following sections the ways in which autophagy can be both tumorigenic and/or tumor suppressive (Figure 3).

Recently published data indicate that the role of autophagy in cancer depends on a specific context. Autophagy may function as a tumor-suppressive mechanism during early tumorigenesis, but its role in advanced cancer remains unclear. Direct evidence shows that the tumor suppressor function in autophagy comes from the fact that some ATG-proteins, such as Beclin-1, exhibit anti-oncogenic functions. Inactivation of autophagy-related genes, such as *Beclin-1*, leads to increased tumorigenesis in mice while the overexpression of these genes (*Beclin-1*, *Atg5*) inhibit the formation of human breast tumors in mouse models [49]. The tumor suppressor function of *Beclin-1* is supported by the genetic evidence that *Beclin-1* is mono allelically deleted in breast, ovarian, and prostate tumors [50,51]. Distinct to the role of autophagy in early tumorigenesis, it is also now widely accepted that autophagy is required for the survival of established cancers [52,53]. In this regard, autophagy inhibitors could be useful as anticancer therapeutic agents [54]. However, the regression of tumor xenografts derived from a large number of human cancer cell lines is not detected upon inhibition of autophagy [55]. However, the autophagy inhibitor, chloroquine (CQ), suppressed the growth of cancer cell lines, even if its effect is autophagy-dependent remains elusive.

### 2.4. Autophagy as Tumor Suppressor

Autophagy is an equilibrating cell process that when disturbed, can support and speed the tumor progression. Autophagy operates as an antitumor mechanism by the elimination of unhealthy intracellular parts and/or proteins, thereby fixing the cell homeostasis in relation to cell growth arrest and genome integrity disturbance [56]. Autophagy may also protect and support tumorigenesis initiation by the limitation of necrosis and chronic inflammation, which are associated with the release of proinflammatory HMGB1 [57].

Recent available data indicate that the leading role of autophagy in cancer cells is to increase the stress tolerance of cells, which is used to maintain tumor cell survival [58]. Lately, many scientists showed that, even when there are sufficient nutrients, some mutations like the ones in H-ras or K-ras act like autophagy triggers in human cancer cell lines [59]. The elimination of the main key proteins of autophagy in these cells was shown to stop the growth of cells, confirming that autophagy rules tumor cell survival, which suggests that the inhibition of autophagy in tumors with high levels of autophagy, specifically the ones with Ras proteins, is an efficient approach for developing new successful treatments. 

### 2.5. Autophagy in Tumor Promotion or Progression

More evidence from current published research data indicates that the eminent function of autophagy in tumor cells is to provide them abilities to overcome induced stress, and therefore helps to keep tumor cell alive [60]. Inactivation of autophagy genes that are predominantly active during tumor progression has successfully been shown to allow the induction of cell death [61]. Increased proliferation in cancer cells correlates with increased energetic and metabolic demands. From in vivo experiments, exposure to metabolic induced stress was shown to impair survival in autophagy-deficient cells compared to autophagy-proficient cells [62]. We previously discussed how low levels of oxygen and lack of nutrients, which are the most representative of metabolic stresses, can activate autophagy and therefore help to maintain cellular biosynthesis and survival. Hypoxia pathways in the tumor are known to raise the production and expression of angiogenesis factors, such as vascular endothelial growth factor (VEGF), platelet-derived growth factor (PDGF), and inducible nitric oxide synthase (iNOS) [24]. Hypoxia shapes the tumor microenvironment, especially by the attraction of immune cells that will provide after-activation essential factors for tumor growth and expansion. Autophagy was found to be abnormally elevated in human pancreatic cancer cell lines, and also in tumor specimens. These cells promote tumor growth by keeping the production of energy stabilized. The inhibition of autophagy in these cells leads to tumor regression and extended survival (Figure 3).

### 2.6. Targeting Autophagy for Cancer Prevention

Due to the fact that autophagy plays a crucial role in the inhibition of tumor growth, the initiation of autophagy can be a promising way to prevent cancer. The pi3k-Akt-mTOR signaling pathway is often not regulated in human solid tumors, and the suppression of the mTOR pathway can induce autophagy. One of the famous mTOR inhibitors, rapamycin, reduces 90% of induced lung tumors in murine models. [63,64]. Traditional herbal medicine extracts had also shown abilities that reduce the proliferation of cancer cells by the induction of autophagy. However, several studies showed the role of autophagy in chemotherapy resistance by induction of cell survival.

Nevertheless, the suppression of autophagy to increase the efficiency of anticancer therapies is another alternative, as autophagy is mostly higher in both tumor cancer cells and healthy normal cells exposed to cancer therapies [65,66].

## 3. Tumor Microenvironment

**Figure 4:**
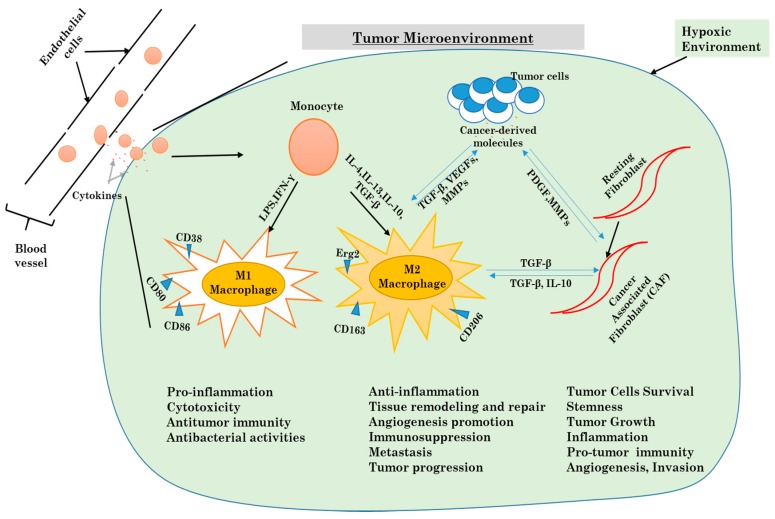
Mechanisms underlying the differentiation of macrophages and fibroblasts in the tumor microenvironment.

For many years, cancer studies and cancer-related research were focused only on cancer as being limited just to cancer cells, ignoring the environment created in the tumor. Cancer was thus believed to be just a disease characterized by a cell-autonomous process. However, it has been acknowledged that tumors are heterogeneous organs, made of a various number of stromal components which are crucial players and not just participants in the tumorigenic process. One of the interests of cell laboratories is to investigate the role of cancer-associated macrophages, cancer-associated fibroblasts, and the extracellular matrix components that they secrete [67]. Obtaining a much better understanding of the usual molecular pathways involved in the interactive bi-directional communication between tumor components and stroma cells will help researchers to manipulate the tumor microenvironment (Figure 4).

### 3.1. Cellular Components of the Tumor Microenvironment

The tumor microenvironment is a confusing, intriguing and difficult system to study. Various stromal cells, including tumor-associated macrophages, cancer-associated fibroblasts (CAFs), endothelial cells, other immune cells like T lymphocytes, adipocytes cells, mesenchymal stems cells (MSCs), and various produced cytokines are major components [68].

Alternatively activated macrophages and cancer-associated fibroblasts display several pro-tumorigenic functions that have important roles in tumor development and progression, such as the ability to provide cytokines and induce tumor angiogenesis and invasion [69] (Figure 4).

Inflammation was one of the first biological processes to be suspected of having a strong connection with tumor development. It was the first time that components other than cancer cells were being investigated with regards to their support in tumor progression. The link between inflammation and cancer was later associated with the presence of a high number of leukocytes in tumor tissues. Today it is recognized that the chronic inflammation plays a major rule in tumor initiation, settlement, and in the invasion of other body sites [70].

Various publications had pointed out the role of each component, particularly TAMs, CAFs and endothelial cells, in the promotion of tumor growth and the progression of cancer. Instead of fighting against cancer like most other cells in the tumor microenvironment, M2 macrophages are polarized by cancer-derived factors to promote tumor growth, immunosuppression, and metastasis. Therefore, the knowledge of pathways involved in the maintenance and control of the tumor microenvironment will provide a better-improved understanding of cancer biology, and open new opportunities for much more specific targets for cancer therapy [71,72].

### 3.2. Inflammatory Response and Autophagy in the Tumor Microenvironment

Inflammatory mediators are often highly expressed in a tumor microenvironment. It is now very known that chronic inflammation participates enormously in tumorigenesis. Tumor-derived molecules are responsible for the activation of inflammatory cells like macrophages and fibroblasts. Macrophages in the tumor microenvironment come from the differentiation tumor-resident macrophages and from monocytes recruited from blood vessels. The differentiated macrophages are then polarized in tumor-associated macrophages. There are two known phenotypes for TAMs, which are M1 macrophages (pro-inflammatory macrophages) and M2 macrophages (anti-inflammatory macrophages) [73]. As for cancer-associated fibroblasts, they are obtained from normal fibroblasts already present in the tissue. The CAFs acquire specific characteristics similar to myofibroblasts. Both TAMs and CAFs are the most present cells in the tumor microenvironment. Various studies have highlighted how both cells play a crucial role in tumor ignition, progression, evasion, and resistance to chemotherapy [74]. 

In the tumor microenvironment, TAMs and CAFs are involved in the evolution of a tumor by the production of cytokines. Some cytokines are common to both cells, such as IL-6, IL-8, IL-10, and IFN-γ, but they also produce different cytokines [75]. Cytokines play a major role in the development of chronic inflammation and in the anti-tumor response, but also participate in all steps of cancer progression via inflammation [76]. In addition to the production of keys mediators for tumor growth, inflammation can activate autophagy [77]. 

#### 3.2.1. Autophagy and Macrophages

Hematopoietic stem cells (HSCs) stay in the bone marrow where they are in a dormant and non-differentiated state. In response to particular stimuli, they can differentiate into the various type of blood cells [78]. As blood cells have a short life period, they have to be replaced consistently; therefore a constant differentiation of HSCs is required. The bone marrow environment is hypoxic and as shown above, hypoxia is one of the main initiators of autophagy. Evidence from recent studies shows that autophagy is essential for both self-renewal and differentiation of HSCs [79]

Chemokines like CCL2 attract monocytes in the tumor microenvironment and later differentiate into TAMs [80]. It has been shown that CCL2 can prevent apoptosis in monocytes through the upregulation of antiapoptotic proteins, and Roca H and al. noticed an excessive activation of autophagy in monocytes; therefore, we can conclude that autophagy is highly implicated in the recruitment of monocytes [81]. 

CSF-1 is known to be the main cytokine involved in the differentiation of macrophages from monocytes when they migrate in tissue after extravasation. After stimulation of monocytes by CSF-1, autophagy is activated by the phosphorylation of ULK1 [82,83]. Additionally, differentiation of monocytes by CSF-1 requires the activation of caspases, more specifically caspase-3 and caspase-8, through regulation of the Akt pathway [84]. CSF-2, another stimulating factor for monocytes, can also initiate the differentiation of monocytes to macrophages via the MAPK/JNK pathway by preventing the interaction between BECN1 and BCL-2, which triggers autophagy [85]. 

As mentioned above, TAMs can be divided into two distinct phenotypes, which are M1 macrophages that are more active in the Th1-mediated response against microorganisms, and M2 macrophages that act more like tissue healer. Several studies have described the vital function of the NF-kB pathway in macrophage polarization and how it is the linking bridge between inflammation and cancer [86,87,88]. The mTOR, as cited above, is a key kinase protein in the regulation of autophagy, but also participates in the polarization of monocytes into TAMs. As shown above, inhibition of caspase-8 with CCL2 and IL-6 has the ability to suppress apoptosis, promote autophagy and therefore increase M2 macrophage polarization. 

Altogether, these findings determine the essential role of autophagy in all steps of TAMs production from HSCs differentiation, the recruitment of monocytes, the differentiation of monocytes to macrophages and the polarization of macrophages into TAMs.

#### 3.2.2. Autophagy and Fibroblasts

In recent years, many studies had been conducted in order to understand the role played by autophagy and CAFs in the tumor microenvironment. CAFs are differentiated from normal resident fibroblasts, and actively contribute to the progression of different cancers by the initiation of tumor growth and angiogenesis. Ubaldo E Martinez-Outschoorn et al. showed that the co-culture of fibroblasts with breast cancer cells creates a microenvironment rich in inflammatory mediators and growth factors (IL6, IL-8, IL-10, MIP1α, IFNγ, RANTES, GM-CSF, TGFβ2, FGFβ, MMP2, MMP9, fibronectin, and activated PAI-1] in what they described as a “cytokine storm”. They also discovered that autophagy in the tumor microenvironment is associated with an inflammatory response, and that the mediators of inflammation are enough to activate autophagy. However, they mentioned that hypoxia alone is not enough to initiate the inflammatory response observed during their experience [89].

His team was able to demonstrate to the role of the Cav-1 protein (a principal structural component of caveolae, specialized plasma membrane invaginations involved in the regulation of several cellular processes, including the control of cell signaling) and the NFkB pathway in the induction of autophagy in CAFs in the tumor microenvironment [90]. 

Meanwhile, Claudia Capparelli et al. showed that a protein member of the BH-3-only subfamily of Bcl-2 family proteins, BNIP3, was overexpressed in fibroblasts during autophagy with a loss of Cav-1 expression, but increased the expression of Beclin1 and ATG16L1, key markers of autophagy. Fibroblasts with a high expression of BNIP3 accelerated tumor growth, while overexpression of ATG16L1 showed constitutive activation of autophagy and increased tumor growth [91].

Numerous studies indicate that autophagy is widely implicated in the regulation of the inflammatory response in tumor microenvironment through TAMs and CAFs. These cells provide clear evidence that autophagy functions as a key moderator for macrophage production, by modulating HSC maintenance, monocyte differentiation into macrophages, and monocyte/macrophage recruitment, as well as for macrophage polarization. Autophagy also controls the differentiation of fibroblast into CAFs, therefore, it is the central piece of the immune response in the tumor microenvironment. 

## 4. Conclusions and Perspectives

Mechanisms behind the interaction between autophagy and the components of the tumor environment still remain unclear and need to be explored. Currently, we know that bioactive molecules such as cytokines, growth factors, and chemokines produced during inflammation by tumor infiltrating cells, sustain cell growth, limit apoptosis, and promote new blood vessel formation [92]. Macrophages play a key role in therapeutic resistance to chemotherapy, and the inflammatory mediators can also initiate autophagy [93]. 

As discussed in this review, the tumor microenvironment can activate autophagy through the creation of a suitable environment for autophagy initiation by tumor stromal cells, more specifically TAMs and CAFs, but we also saw that autophagy can modify the tumor microenvironment through the synthesis of new blood vessels in tumors, the supply of nutrients, and the shaping of responses to inflammation by interaction with stromal cells. This is one side of autophagy function that helps the cancer cells in the tumor microenvironment to overcome metabolic stress, maintain homeostasis, and survive in the poor physical environment. Although many autophagy-related therapies had already proven to be efficient, it is hard to state that targeting autophagy is a trustful way of curing cancer, as it has dual roles, as shown above. Much-needed investigations on how the autophagy together with TAMs and CAFs shape the tumor microenvironment are still needed. A clear knowledge of pathways involved in tumor fate due to the activation of autophagy, and alternatively polarized macrophages and fibroblasts will certainly open new insights into cancer, and potentially provide new specific targets for cancer therapy. 

## Abbreviations

AMBRA1	Autophagy and beclin 1 regulator 1
AMPK	AMP-activated protein kinase
APC	Antigen-presenting cell
ATG	Autophagy-related genes
Bcl-2	B-cell lymphoma 2
BINP3	BCL2 Interacting Protein 3
CAFs	Cancer-associated fibroblasts
Cav-1	Caveolin-1
CCL 2	Chemokine (C–C motif) ligand 2
CD	Cluster of differentiation
CQ	Chloroquine
DC	Dendritic cells
ECM	Extracellular matrix
FGF	Fibroblast growth factor
FIP200	FAK-family interacting protein of 200 kDa
GM-CSF	Granulocyte macrophage colony-stimulating factor
HCQ	Hydroxychloroquine
HIFs	Hypoxia-inducible factors
HMGB1	High mobility group box 1
HSCs	hematopoietic stem cells
IFN	Interferon
IL	Interleukin
PI3K	Phosphoinositide 3-kinase
JNK	c-Jun N-terminal kinases
LAMP	Lysosomal-associated membrane protein
LC3	Light chain 3
MAPK	Mitogen-activated protein kinase
MEC	Mammary epithelial cell
MIP	Macrophage inflammatory protein
MMP	Matrix metalloproteinase
MSCs	Mesenchymal stem cells
mTOR	Mammalian target of rapamycin complex
mTORC1	Mammalian target of rapamycin complex 1
NF-κB	Nuclear factor kappa B
PARP	Poly-ADP-ribose polymerase
PDGF	Platelet-derived growth factor
PE	Phosphatidylethanolamine
RAGE	Receptor for advanced glycation end product
RANTES	Regulated on Activation, Normal T Cell Expressed and Secreted
ROS	Reactive oxygen species
SNAP29	Synaptosome Associated Protein 29
TAMs	Tumor-associated macrophages
TGFβ	Transforming growth factor beta
TNF-α	Tumor necrosis factor-α
ULK1	Unc51-like kinase 1
VAMP8	Vesicle associated membrane protein 8
VEGF	Vascular endothelial growth factor
